# Using patient-reported outcome measures to assess the effectiveness of social media networking programs for people living with overweight and obesity to adopt a healthier lifestyle

**DOI:** 10.3389/fpubh.2023.1161851

**Published:** 2023-06-12

**Authors:** Alend Saadi, Jean-Marc Fellrath, Joanna Bec-Moussally, Chrysoula Papastathi-Boureau, Céline Blanc, Valentin Courtine, Léo Vanini, Marc Marechal, Florence Authier, Bertrand Curty, Pierre Fournier, Michele Diana, Stéphane Saillant

**Affiliations:** ^1^Department of Surgery, Neuchâtel Hospital, Neuchâtel, Switzerland; ^2^Faculty of Biology and Medicine, University of Lausanne, Lausanne, Switzerland; ^3^Faculty of Medicine, University of Geneva, Geneva, Switzerland; ^4^Ligue Pulmonaire Neuchateloise, Neuchâtel, Switzerland; ^5^Espace Nutrition, Neuchâtel, Switzerland; ^6^Centre Médical de La Côte, Corcelles, Switzerland; ^7^Service de Chirurgie, Hôpital de Nyon, Nyon, Switzerland; ^8^Service de Chirurgie Viscérale, Centre Hospitalier Universitaire Vaudois (CHUV), Lausanne, Switzerland; ^9^IRCAD, Research Institute Against Digestive Cancer, Strasbourg, France; ^10^Department of Surgery, Strasbourg University Hospital, Strasbourg, France; ^11^Département de Psychiatrie Générale et de Liaison, Centre Neuchâtelois de Psychiatrie, Neuchâtel, Switzerland

**Keywords:** obesity, overweight, PROM, social media, lifestyle, internet, quality of life (QoL)

## Abstract

**Introduction:**

Overweight, obesity, and their associated health complications have become a major public health issue. Online approaches have been rarely attempted to address the problem. The aim of this study was to evaluate the effectiveness of using social media networking for people living with overweight and obesity to adopt a healthier lifestyle with a three-month multidisciplinary healthcare program. Effectiveness was assessed through questionnaires on patient-related outcome measures (PROMs).

**Materials and methods:**

Two non-profit associations designed the program delivered to people living with overweight and obesity in a closed group via Facebook, the popular social network. The three-month program had three main axes, namely nutrition, psychology, and physical activity. Anthropomorphic data and sociodemographic profiles were collected. Quality of life (QoL) was assessed at the beginning and at the end of the intervention using PROM questionnaires for six different domains, i.e., body image, eating behavior, physical, sexual, social, and psychological functioning.

**Results:**

Six hundred and twenty persons participated in the program; 567 persons consented for the study, and 145 completed the questionnaires entirely. QoL was significantly improved in five out of six domains, i.e., body image, eating behavior, as well as physical, sexual, and psychological functioning. The improvement was valid regardless of age, gender, initial body mass index, person with or without children, educational level (primary versus secondary versus high school), and occupation (employment compared to unemployment or any kind of social assistance). In multivariate analysis, living as a couple was an independent factor correlated to a positive progression in four domains, i.e., body image, eating behavior, as well as physical, and psychological functioning.

**Conclusion:**

This study showed that an online lifestyle intervention might be a promising way of improving the quality of life of people living with overweight or obesity.

## 1. Introduction

Obesity is a chronic and progressive condition with a significant biological predisposition (1). Overweight and obesity are associated with many complications, including cardiovascular, metabolic, respiratory, and osteo-articular problems (2). Its prevalence keeps increasing worldwide as it has become a major public health issue (3). The links between obesity and mental health are unclear. However, it seems obvious that there is a major mutual influence. When body dissatisfaction is present, it could negatively affect psychological health and physical activity, leading to dysfunctional eating behaviors. Consequently, obesity can also have a major impact on the mental health of patients (4–6). There are multiple treatments for obesity. However, the use of strict low-calorie diets. Additionally, popular diets without specific calorie targets have shown its limitations, since they lead to weight regain in most cases (7–9). Beyond prevention and once the disease has been confirmed, it initially requires multidisciplinary treatments focused on lifestyle changes to promote healthier habits (10). However, due to the disease’s complex and multifactorial origin, the implementation of such projects is uneasy (11). Additionally, at community level, it requires substantial resources to treat a large number of people (12).

Implementing health interventions via the Internet and social media networking have the great advantage of treating large numbers of persons at reduced costs (13). When interactive, it allows the creation of a dynamic group with participants, which has been associated with better results. It is a component of the social cognitive theory, which suggests that prior to self-efficacy, social support enhances the process of behavioral change and maintenance (14). The implementation of online health projects via the Internet as a large-scale prevention and treatment resource could very well represent a major opportunity to handle overweight and manage obesity as a complement to conventional face-to-face treatments.

Several online programs have already been set up. The results are heterogeneous, probably due to different methods and target populations; nevertheless, they remain promising (15–19). One of the issues is the methods used for the assessment of their effectiveness by measuring weight loss or maintenance. Experience has shown that lifestyle interventions led to modest weight loss (10). Additionally, overweight and obesity have multiple consequences on quality of life (QoL), and more particularly so in psychological, physical, and social domains. The World Health Organization (WHO) defines QoL as individuals’ perception of their position in life within the context of the culture and value systems in which they live and in relation to their goals, expectations, norms, and concerns (20). It is why, beyond any potential weight loss, it is crucial to evaluate the QoL of participants, as it has not been achieved effectively so far. In addition, the way that QoL is measured is of paramount importance. It appears utterly necessary to use appropriate tools to assess it effectively. First, it is essential to include patient opinions through questionnaires on patient-reported outcome measures (PROMs) (21). PROMs are tools used to measure patient perceptions of their own health status, clinical outcomes, mobility, and quality of life. The use of patient-reported indicators helps to provide a more comprehensive picture of health treatment performance (22). Additionally, disease-specific PROMs seemed more effective and sensitive in gauging outcomes than generic QoL questionnaires (23).

The aim of this study was to evaluate the effectiveness of a multidisciplinary health program for people living with overweight and obesity using PROMs questionnaires delivered via Facebook, the social media network, by two Swiss-based non-profit organizations (NPOs).

## 2. Materials and methods

### 2.1. Program description

Two non-profit organizations have launched a health program for people living with overweight and obesity (24–26) in the Neuchâtel canton in Switzerland. It was a three-month intervention prepared by a multidisciplinary team of professionals in order to promote the adoption of healthier lifestyle habits. The inclusion criteria were as follows: place of residence in the Neuchâtel canton, to be aged over 17 with a body mass index (BMI) ≥25 kg/m^2^. The BMI was calculated according to the declared weight and height of the participants. There were no exclusion criteria other than a BMI <25 kg/m^2^ and the geographical component for operational reasons. This was the decision and will of the promoting organizations to be as inclusive as possible, targeting large public health interventions. Involvement was charge-free for participants. Registrations began following a press conference for the local traditional press on August 15, 2021 along with an announcement on social media networks. The program was delivered from September 5 to December 5, 2021.

### 2.2. Implementation

The program was designed in the form of posts with either short written messages and/or didactic videos. Field professionals developed all educational materials and recommendations, and a team of experts dedicated to the management of obesity reviewed and validated it. Community managers published the posts usually once a day during weekdays and twice to three times on weekends. These managers moderated the debates between attendees, and they were also available to answer questions or refer to specific enquiries to the healthcare professionals in charge of each domain. Some face-to-face events were also organized upon registration to allow participants to meet for activities such as outside walking and mindful eating. Table 1 summarizes the topics covered, their domains (general information, nutrition, physical activity, or psychology), their format (text or video), as well as the overall and week-by-week chronology.

**Table 1:** Summary of the program.

P	F	W	Publication title	P	F	W	Publication title
1	T	Su	Welcome.	66	V	Mo	Recovery after sport – stretching?
2	V	Su	Beginning of the program.	67	T	Tu	Evaluating emotions that drive to eat.
3	V	Mo	Why we are NOT going on a diet.	68	T	We	Welcome to Kathmandu.
4	V	Tu	We take our measurements.	69	T	We	Nepalese cuisine.
5	T	Tu	How to follow this lifestyle change program?	70	T	Th	Messages for men.
6	V	We	Photographing your dishes.	71	T	Fr	Back to emotions.
7	T	Th	Let us move (Install a pedometer)	72	V	Sa	Positive Anchor. What will yours be?
8	T	Th	Technical aspects.	73	T	Su	Questions & Answers.
9	T	Fr	Today, we sort our clothes.	74	T	Su	Sunday evening discussion.
10	T	Fr	Charter of good conduct.	75	V	Mo	Upper body exercises.
11	T	Sa	Food and digestion.	76	T	Mo	Preparing for the walk.
12	T	Sa	A Picture Worth a Thousand Words.	77	T	Tu	Sugar.
13	T	Su	Day off, we walk and get some fresh air.	78	V	We	A huge BRAAAAAVO. Welcome to Beijing.
14	V	Su	Sunday evening discussion.	79	T	Th	The guilt.
15	T	Mo	Benefits of walking.	80	V	Fr	Fight and Flight.
16	T	Mo	How many steps this Monday?	81	V	Sa	We step on the scale – assess energy level.
17	V	Tu	The well-balanced meal – Part 1.	82	T	Su	Sports exercise (hide scale for 1 month).
18	V	We	Walk to Beijing. Our great Odyssey.	83	T	Su	Sunday evening discussion.
19	T	Th	Plan things!	84	T	Mo	The stairs to shape lower body part.
20	T	Th	Dietetic workshop.	85	V	Tu	Challenge: one week without sugary drinks.
21	V	Fr	The well-balanced meal – Part 2.	86	V	We	Eat mindfully.
22	T	Fr	Analysis of your dishes (photographs).	87	V	Th	Are you eating enough fiber?
23	T	Sa	Diaphragm breathing.	88	T	Fr	Constipation, list of high fiber foods.
24	T	Su	Benvenuti a Milano.	89	V	Sa	How does breathing work?
25	T	Su	Today we cook Italian.	90	T	Su	Game – simple things that make us happy.
26	T	Su	Sunday evening discussion.	91	T	Su	Sunday evening discussion.
27	V	Mo	We all do squats with the coach.	92	T	Mo	8,708 daily steps (group average)
28	T	Mo	Dietary workshops.	93	T	Mo	We walk together.
29	T	Tu	Physiological or psychological hunger?	94	V	Tu	Eating behaviour – behavioural strategy.
30	V	We	Welcome to Athens.	95	T	We	Welcome to Kyoto – Praise of simplicity.
31	T	We	Psychiatric follow-up what it is?	96	T	Th	Body Image.
32	V	Th	How to change your habits?	97	T	Th	The negative body image.
33	V	Fr	Am I hungry or is it a desire?	98	V	Fr	“Healthy” foods not always good!
34	V	Sa	Stress relief exercise.	99	T	Sa	Are my thoughts about my body biased?
35	T	Su	Walking 30 min a day.	100	T	Su	Sunday’s big game – Everything is linked.
36	T	Su	Sunday evening discussion.	101	T	Su	Sunday evening discussion.
36	V	Mo	Increase the basal metabolic rate – walking.	102	T	Mo	7 day squat challenge.
38	T	Tu	Breakfast or a snack in the morning?	103	V	Tu	The Vicious Circle of Body Checking.
39	V	We	Welcome to Red Square in Moscow.	104	T	We	Sweeteners.
40	T	We	We cook Russian.	105	T	Th	Learn to appreciate yourself.
41	V	Th	The vicious circle of diets.	106	T	Fr	Let us observe the quality of our sleep!
42	T	Fr	How long should a meal last?	107	V	Sa	Hourglass
43	V	Fr	Eating slowly	108	T	Sa	We relax outside, in nature!
44	V	Sa	Install the self-observer	109	T	Su	Day off – The Afghan March.
45	T	Su	Day off, so take care of ourselves.	110	T	Su	Sunday evening discussion.
46	T	Su	Sunday evening discussion.	111	V	Mo	Coach’s program for this week.
47	V	Mo	Without hunger, a craving for food is brief.	112	T	Tu	Menopause.
48	T	Tu	Satiety and satiation. When stop eating?	113	V	We	Fast food – junk food.
49	V	We	We arrive today in Tehran.	114	T	Th	Quality of our sleep. Analysis of our sleep.
50	T	We	Iranian cuisine.	115	T	Fr	Strategies to get back to sleep well step 1.
51	T	Th	Strategies for not nibbling.	116	V	Sa	Posture and breathe meditation exercise.
52	T	Th	Dietary workshop.	117	T	Su	Walking in the rain.
53	T	Fr	Accept your own emotions.	118	T	Su	Sunday evening discussion.
54	V	Sa	Set up a break.	119	T	Mo	Physical activity over the long term?
55	T	Su	Sunday, our day to us!	120	V	Tu	Sharing the ideal meal with the dietician.
56	T	Su	Sunday evening discussion.	121	T	We	Strategies to get back to sleep well step 2.
57	V	Mo	The climbers.	122	T	Th	Review of the main dietary messages.
58	V	Tu	Emotions can affect the weight.	123	T	Th	Diet Workshops – Summary
59	T	We	Our daily steps lead us to India.	124	T	Fr	Maintain your lifestyle change.
60	T	We	We cook Indian.	125	V	Sa	We take our measurements.
61	V	Th	Healthy snacks.	126	T	Su	Program evaluation.
62	T	Fr	What type of eater are you?	127	V	Su	Well done and thank you very much.
63	V	Sa	Include a daily ritual.				
64	T	Su	Day off – Pick-up endorphins.				
65	T	Su	Sunday evening discussion.				

Column P represents the post number. Column F indicates the format of the post: text (T) versus video (V). Column W indicates the weekday when the post was published, and the colors indicate the alternating weeks. The last column indicates the title of the post, as well as the domain: yellow for general information, orange for physical activity, blue for psychology, and green for nutrition.

### 2.3. The three main axes of the program

**Physical activity:** A professional sports coach was in charge of this aspect. The aim was to find a daily or weekly routine habit to move and do exercises. A pedometer was provided. The participants were asked to report the count of their daily steps. The project revolved around the symbolic objective of an odyssey to reach Beijing (China) on foot from Neuchâtel (Switzerland) counting the steps of all participants during the 3 months. Adapted fitness exercises, including several levels of difficulty to be accessible to all, were shown and encouraged with videos.

**Nutrition:** The first objective was to break the vicious circle of repetitive restrictive low-calorie diets that many candidates previously experienced. A dedicated dietician prepared the posts. The objectives were to allow attendees to reconnect with their appeased food sensations and to eat with pleasure. Another point was to promote a healthy diet according to the recommendations of the Swiss Society of Nutrition (27). Healthy recipes from around the world were featured as the symbolic walking odyssey progressed through different countries.

**Psychology:** A dedicated psychologist prepared many topics to discuss with participants. The objective of this axis was to help participants to understand the psychological mechanisms underlying the unhealthy habits and behaviors. First, general information about the notion of motivation, and how to plan any behavioral change were delivered. Participants were then taught how their emotions could influence weight loss or gain, especially through their eating behaviors. They were asked to train to identify and regulate their emotions. A special focus was put on stress management, guilt decrease, and emotional acceptation. Afterwards, the link between sleep disorders and weight gain was clarified and behavioral strategies were mentioned to improve sleep quality. Finally, negative body image has been dealt with through posts explaining the role of developmental factors such as pressures to reach occidental beauty standards usually conveyed by the media, past interpersonal experiences, morphological characteristics, cognitive distortions, checking and avoidance behaviors in the maintenance of body dissatisfaction. Throughout this axis, change was encouraged through self-observational and exposition exercises (28) to reach the objective of a better lifestyle.

A dedicated platform was created for the program, allowing for the secure registration of participants protected via an individual password. Personal data and registration forms were collected directly through this secured online platform in accordance with the Swiss regulation (ISO 27001 certification).

Once registered, participants were able to join a closed group on Facebook. The animation was delivered via this channel to promote group dynamics and mutual aid between participants.

The global costs of the program were estimated at 130,000 Swiss Francs (CHF). Table 2 summarizes the costs. As some items will be used for future editions and other activities, the weighted cost was estimated at CHF 100,000. The costs were entirely financed by non-profit organizations. They financed themselves through their own remunerated activities, donations, and members’ subscriptions. The program was charge-free for participants.

**Table 2:** Summary of program costs.

Items	Estimated costs (CHF)
Preliminary preparations	20,000
Information technology	35,000
Dedicated activities of obesity healthcare professionals	50,000
Administration and management	15,000
Others	5,000
Volunteer work	10,000
Total	135,000

CHF: Swiss Francs.

At inclusion, participants completed a questionnaire with items related to their anthropomorphic and sociodemographic characteristics such as weight, height, waist circumference, gender, age, educational level, family status (living in a couple or being single, person with children), occupation, and place of birth. They were also asked to complete questionnaires about their QoL, health condition, and expectations at the start and at the end of the program. The QoL questionnaires used included the Body-Q PROMs specific to obesity treatment (29). Six different domains were measured, i.e., body image, eating behavior, as well as physical, sexual, social, and psychological functioning. Each domain was evaluated with 5 to 10 questions and could be used independently. The initial score obtained was then converted into a score ranging from 0 to 100. Higher scores reflected a better outcome, except for the physical function. This last one was more a measure of physical disability. In case of improvement, the score dropped unlike the other domains.

### 2.4. Study population and design

All the data came retrospectively from the program described above and called “Neuchâtel s’attaque au surpoids” (“Neuchâtel tackles overweight”). It is why the criteria for participation, inclusion, and exclusion are by extension similar to those of the studied program.

The program ran from September 5 to December 5, 2021. The anthropomorphic data and sociodemographic profiles of the participants at the start and at the end of the program, as well as the QoL PROMs were available. Only the participants who consented to the use of their data for scientific purposes were examined. We assessed changes in QoL, as well as weight loss and waist circumference between the beginning and the end of the program. For this step, only data from participants who entirely provided the data and completed questionnaires were considered.

## 3. Statistical analyses

The statistical analyses were performed using the R software. The analyses on the effect of the program were conducted using Student’s parametric tests for paired samples. The scores obtained in the QoL, weight, and BMI at the beginning and at the end of the program were compared. If the difference was significant, a multiple linear regression was applied to the differences between the values on the final balance sheet and those on the initial balance sheet. The objective of this multiple linear regression was to determine whether gender, age, marital status, presence of children, occupation, educational level, and BMI were related to the evolution of the QoL. This procedure was automated by the glmulti function (30) to fit the best model based on the Bayesian Information Criterion (BIC) (31). The statistical significance threshold was set at *p* ≤ 0.05. Afterwards, we calculated whether the evolution of the QoL varied according to anthropomorphic data and sociodemographic profiles registered at the start of the program.

For the QoL domains which showed significant improvement, a multivariate analysis was performed. The following parameters were considered: age and baseline BMI as linear parameters, followed by gender, family status (living as a couple or being single), persons with or without children, educational level (primary versus secondary or high school), and occupation (employment compared to unemployment or any kind of social assistance), as well as country of birth (Switzerland versus another country) as binary parameters.

## 4. Results

**Figure 1:**
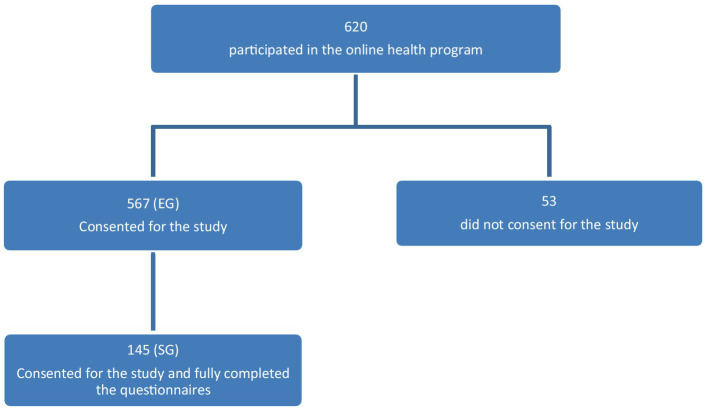
Inclusion process. EG, entire group; SG, study group.

Six hundred and twenty persons subscribed to the program; 567 of them gave their consent for this study. These respondents made up the entire group (EG). Among the 567 persons, 145 had fully completed the QoL questionnaires at the start and at the end of the program. These 145 persons made up the study group (SG). Figure 1 summarizes the inclusion process. Table 3 summarizes the characteristics of EG whereas Table 4 summarizes those of the SG.

**Table 3:** Characteristics of the entire group.

	25 ≤ BMI < 30		30 ≤ BMI < 35		35 ≤ BMI		Total	
Nb of participants	197	*100%*	214	100%	156	*100%*	567	*100%*
Women	144	*73.1%*	145	67.8%	117	*75%*	406	*71.6%*
Men	53	*26.9%*	69	32.2%	39	*25%*	161	*28.4%*
BMI (kg/m^2^)	27.5 (1.4)		32.4 (1.5)		38.6 (3.4)		32.4 (4.8)	
Age (years)	48.7 (12.8)		49.6 (13.0)		48.4 (11.9)		49.0 (12.6)	
Comorbidities								
Diabetes	5	*2.5%*	24	*11.2%*	18	*11.5%*	47	*8.3%*
Hypertension	30	*15.2%*	61	*28.5%*	47	*30.1%*	138	*24.3%*
Sleep apnea	15	*7.6%*	40	*18.7%*	40	*25.6%*	95	*16.8%*
Tobacco use	29	*14.7%*	28	*13.1%*	26	*16.7%*	83	*14.6%*
Country of birth								
Switzerland	157	*79.7%*	177	*82.7%*	126	*80.8%*	460	*81.1%*
Other(s)	40	*20.3%*	37	*17.3%*	30	*19.2%*	107	*19.9%*
Civil status								
In a couple	112	*57.5%*	133	*62.2%*	77	*49.3%*	322	*56.8%*
Single	36	*18.3%*	36	*16.8%*	38	*24.4%*	110	*19.4%*
Divorced	45	*22.8%*	40	*18.7%*	38	*24.4%*	123	*21.7%*
Widower	4	*2.0%*	5	*2.3%*	3	*1.9%*	12	*2.1%*
Children	41	*20.8%*	42	*19.6%*	42	*26.9%*	125	*22.0%*
Professional situation								
Employed	142	*72.1%*	154	*72.0%*	105	*67.3%*	401	*70.7%*
Insurance (invalidity, unemployment, pension, social assistance)	38	*19.3%*	49	*22.8%*	41	*26.3%*	128	*22.6%*
At home	14	*7.1%*	7	*3.3%*	8	*5.1%*	29	*5.1%*
Student	3	*1.5%*	4	*1.9%*	2	*1.3%*	9	*1.6%*
Education								
Compulsory schooling	16	*8.1%*	26	*12.1%*	23	*14.7%*	65	*11.5%*
Secondary education	108	*54.8%*	119	*55.6%*	103	*66.0%*	330	*58.2%*
Post-secondary education	73	*37.1%*	69	*32.2%*	30	*19.2%*	172	*30.3%*

Italic data are sample size, percentage of total sample and mean for BMI and age.

**Table 4:** Characteristics of the study group.

	25 ≤ BMI < 30		30 ≤ BMI < 35		35 ≤ BMI		Total	
Nb of participants	51	100%	54	*100%*	40	*100%*	145	*100%*
Women	45	88%	43	*80%*	35	*88%*	123	*85%*
Men	6	12%	11	*20%*	5	*13%*	22	*15%*
BMI (kg/m^2^)	27.5 (1.4)		32.5 (1.4)		38.4 (3.6)		32.3 (4.9)	
Age (years)	50.0 (11.7)		52.7 (12.6)		49.8 (9.5)		51.0 (11.5)	
Comorbidities								
Diabetes	0	*0%*	7	*13.0%*	3	*7.5%*	10	*6.9%*
Hypertension	2	*3.9%*	22	*40.7%*	14	*35.0%*	38	*26.2%*
Sleep apnea	2	*3.9%*	9	*16.7%*	9	*22.5%*	20	*13.8%*
Tobacco use	9	*17.6%*	6	*11.1%*	5	*12.5%*	20	*13.8%*
Country of birth								
Switzerland	48	*94.1%*	49	*90.7%*	33	*82.5%*	130	*89.7%*
Other(s)	3	*5.9%*	5	*9.3%*	7	*17.5*	15	*10.3%*
Civil status								
In union	33	*64.7%*	43	*79.6%*	24	*60%*	100	*71%*
Single	5	*9.8%*	2	*3.7%*	9	*22.5%*	16	*11%*
Divorced	13	*2.,5%*	4	*7.4%*	6	*15%*	23	*15.9%*
Widower	0	*0%*	2	*3.7%*	1	*2.5%*	3	*2.1%*
Children	7	*13.7%*	7	*13%*	10	*25%*	24	*16.6%*
Professional situation								
Employed	40	*78.4%*	34	*63.0%*	30	*75%*	104	*71.7%*
Insurance (invalidity, unemployment, pension, social assistance)	5	*9.8%*	16	*29.7%*	8	*20%*	29	*20%*
At home	5	*9.8%*	4	*7.4%*	2	*5%*	11	*7.6%*
Student	1	*2%*	0	*0%*	0	*0%*	1	*0.7%*
Education								
Compulsory school	3	*5.9%*	4	*7.4%*	4	*10%*	11	*7.6%*
Secondary education	27	*52.9%*	29	*53.7%*	25	*62.5%*	81	*55.9%*
Post-secondary education	21	*41.2%*	21	*38.9%*	11	*27.5%*	53	*36.5%*

Italic data are sample size, percentage of total sample and mean for BMI and age.

Table 5 summarizes the evolution with a univariate analysis of the six domains of QoL at the beginning as compared to the end of the program according to the Body-Q PROMs for the SG. All domains showed significant improvement (*p* < 0.001), except for the domain of social function (*p* = 0.176).

**Table 5:** Quality of life (QoL) score comparative statistics at the beginning and at the end of the program.

	Study group Nb = 145		
	Beginning	End	*p*
Eating behavior (%)	47.2 (9.1)	52.8 (10.8)	0.000***
Physical function (%)	30.6 (14.2)	25.3 (16.6)	0.000***
Sexual function (%)	45.4 (17.7)	49.1 (19.6)	0.000***
Social function (%)	59.0 (15.8)	60.3 (15.3)	*0.176*
Psychological function (%)	48.8 (14.9)	54.6 (16.8)	0.000***
Body image (%)	28.7 (17.9)	37.7 (18.9)	0.000***
Weight (kg)	90.6 (15.7)	88.3 (15.5)	0.000***

Italic data are mean (standard deviation).

For the QoL domains that showed significant improvement, a multivariate analysis was conducted. The parameters of the multivariate analysis are summarized in Table 6. Age and BMI at baseline were included in the model as a continuous numeric variable; the other variables were categorical. In this analysis, we found the following positive correlation:

**Table 6:** Parameters of the multivariate analysis.

Study group	145	*100%*
Gender		
Female	123	*85*%
Male	22	*15%*
Country of birth		
Switzerland	130	*89.7%*
Other(s)	15	*10.3%*
Civil status		
Living as a couple (marriage, cohabitation, civil solidarity pact)	100	*71%*
Single (single, divorced, widower)	45	*29%*
Children		
Yes	24	*16.6%*
No	121	*83.4%*
Professional situation		
Active (employed, student)	105	*72.4%*
Other (invalid, unemployed, retired, social aid, at home)	40	*27.6%*
Education		
Pre-secondary education	92	*63.4%*
Post-secondary education	53	*36.6%*

Italic data are percentage of total sample.

A positive correlation (*p* < 0.01) in favor of people living as a couple compared to single persons was found regarding the improvement in the following domains of the QoL: body image, eating behavior, as well as physical and psychological functioning.

A positive correlation (*p* < 0.01) in favor of female compared to male participants was found for the progression of the physical function QoL domain.

A positive correlation (*p* < 0.01) was found in favor of persons with a lower initial BMI used as a linear parameter for the body image QoL domain improvement.

Finally, the weight and the waist circumference were available at the end of the program for only 46 and 41 participants, respectively. For these, the average weight loss and the average decrease in waist circumference were 6.35 kg and 6.3 cm, respectively.

## 5. Discussion

To the best of our knowledge, this is the first article which specifically assesses the use and effectiveness of a social media-delivered multidisciplinary program open to all adults presenting with overweight or obesity in a geographical area with the primary outcome being the evolution of QoL with PROMs questionnaires. Our major finding is that the QoL of the participants improved (*p* < 0.001) in five out of the six domains studied including body image, eating behavior, as well as physical, sexual, and psychological functioning.

Indeed, various studies have shown the efficacy of an intervention via social media networks but aimed at limited subgroups. In the study by Godino et al. (18), the target group was university students while they were medical students in the study by Krishnamohan et al. (32). Other studies such as Ruotsalainan et al. (33) have focused on teenagers. Youth groups and students were often targeted because they were thought to be more comfortable with technology and social media networking. However, according to recent estimates (34), 58% of the world’s population is connected to the Internet and 45% actively uses social networks. In America and Europe, the Internet penetration rate is 78% and 86% respectively, and the social media use rate is 66% (America) and 55% (Europe) respectively. Facebook is the most used social network according to its own data. Because overweight and obesity are a global community health concern, it is valuable to design projects towards a large proportion of the population. In this study, the median age was 49 years. However, mainly women respond to the offer of treatment for people living with overweight and obesity (16, 18, 19, 33). Our study was no exception with 71.6% of women. Women may suffer from greater social and occupational consequences of obesity, which may lead them to more actively search to lose weight (35).

However, there are studies with larger targets, but with exclusion criteria that we believe to be major and which exclude large sections of the population. On one hand, Hales et al. (19) had the following exclusion criteria: pregnancy, persons outside of the 18–65 age range, unable to attend 3 meetings at the University of South Carolina, psychiatric illness, drug or alcohol addiction, uncontrolled thyroid condition, major health condition, eating disorder, currently participating in a weight loss program. On the other hand, Jane et al. (16) had many exclusion criteria (smoking, lipid-lowering drugs, use of steroids and other lipid-influencing agents, use of warfarin, diabetic persons, thyroid dysfunction, major systemic diseases, gastrointestinal problems, intestinal disorders, proteinuria, liver disease, renal failure, vegetarianism, and in the past 6 months no cardiovascular events, no participation in a clinical trial, and no weight fluctuation). In the present study, the multidisciplinary project was designed to fit as many people as possible. The promoters believed that it was crucial to provide sufficient nuance in dietary and psychological counseling, as well as multiple levels of physical activity exercises to be largely inclusive. Another key point was the daily presence of community managers to answer various questions from participants, and more particularly so from people with health and motility limitations.

The second extremely important point is the use of PROMs questionnaires to evaluate the effectiveness of the program. We know that lifestyle interventions lead to modest weight loss (10) and it is not necessarily a good target. Additionally, BMI does not necessarily adequately reflect the state of health or well-being of people living with overweight and obesity (36). However, most studies via social media networking include BMI as the primary outcome (13–19, 33). The importance of the measurement of the QoL with PROMs is growing (21). In the present study, we found a statistically significant improvement in 5 of the 6 domains measured including body image, eating behavior, as well as physical, sexual, and psychologicalfunctioning.

Social function remained stable but did not progress despite a social life related intervention by addressing issues such as self-esteem and a positive body image. It is likely that 4 months is too short to change social habits. People living with overweight and obesity suffer from ingrained stigma in society. Stigma is sometimes internalized, making social interactions difficult for them. A longer study is probably necessary to observe significant changes (37). Additionally, this aspect has certainly suffered from the post-lockdown effect of the COVID-19 pandemic.

It was also pointed out that most studies on social media networks have been conducted in Western countries and most often involved a well-educated population, suggestive of the difficulty of transposing the results to the whole population (38). However, some studies showed that access to treatment for people living with obesity was not necessarily linked to the sociodemographic profile or educational level (39). This point is also confirmed in the present study. In the multivariate analysis, the improvement of QoL was not significantly correlated to the age, the occupation or the educational level (i.e., pre-secondary versus post-secondary level). This is very encouraging because it suggests that this type of projects could be useful and valid for the majority of the adult population and because overweight and obesity overly affect the underprivileged population. As a result, it is critical to design interventions which fit them.

Financially speaking, the program cost was estimated at 100,000 CHF, which equals to approximately 160 CHF per participant. This is approximately the price of a single consultation with a general practitioner in Switzerland. This program, even if paid for, is most likely financially beneficial to participants and to the health system supervising the management of overweight and obesity given that it is a multidisciplinary comprehensive healthcare program over a period of 3 months.

In addition, many people living with obesity have poor experiences with healthcare professionals and report that they had been stigmatized in that context (40). This type of program in a kind and comprehensive atmosphere in addition to group dynamics may encourage some participants to go further and seek help from dedicated healthcare professionals.

This study has several limitations. The first one originates from the fact that only 145 of the 567 consenting participants entirely completed the QoL questionnaires at the end of the study. This is explained by the fact that entering the program required completing all questionnaires. This action lasted about 30 min. On the other hand, at the end, questionnaire completion was at the discretion of the participants since there was obviously no obligation or consequences for them. Despite the encouragement of the organizers to do so, only a part of the participants also completed the final evaluation questionnaires. As this study retrospectively used the delivered program data, participation and attrition rates were directly related to how it was organized.

Another weakness originates from the fact that there is no comparison group. It is due to the retrospective nature of the study. Additionally, like many obesity treatments, mostly women participated. Finally, the duration of the program was of only 3 months whereas the obesity condition was chronic and relapsing. The program could be considered as an initiation to change for a healthier lifestyle. The promoters made this point clear throughout the program and made participants aware of the need for long-term follow-up by healthcare professionals, especially in case of related complications.

In this study, two major parameters are salient. The first parameter is the broad and inclusive character of the program, and then subsequently the study. Participation in the program was open to all persons aged over 17 living with overweight and obesity without any other exclusion criteria, and particularly health conditions. The second extremely important parameter is the use of PROMs questionnaires to evaluate the effectiveness of the program.

## 6. Conclusion

This three-month multidisciplinary program using a social media network promoting change to a healthier lifestyle for people living with overweight and obesity showed a significant improvement in QoL in five domains including body image, eating behavior, as well as physical, sexual, and psychological functioning. The improvement was irrespective of initial BMI, age, gender, occupation, and educational profile. This may be an additional promising tool to treat large groups of persons suffering from overweight and obesity hand in hand with community conventional treatments.

## Data availability statement

The data analyzed in this study is subject to the following licenses/restrictions: a dedicated platform was created for the program, allowing for the secure registration of participants protected by an individual password. Personal data and registration forms were collected directly through this secured online platform in accordance with the Swiss regulation (ISO 27001 certification). Requests to access these datasets should be directed to marc.marechal@lpne.ch.

## Ethics statement

All procedures performed in this study were in accordance with the ethical standards of the non-profit organizations involved and with the 1964 Helsinki declaration and its later amendments or comparable ethical standards. Informed consent was obtained from all individual participants included in the study.

## Author contributions

AS: substantial contributions to the conception and design, analysis and interpretation of data for the work, and drafting the work. J-MF, JB-M, CP-B, CB, VC, FA, BC, PF, MD, and SS: substantial contributions to the conception and design, interpretation of data, and revising critically the work. LV and MM: substantial contributions to the conception and design, acquisition and analysis of data, and revising critically the work. All authors contributed to the article and approved the submitted version.

## Funding

This study was supported by the two non-profit organizations which delivered the program (22, 23).

## Conflict of interest

The authors declare that the research was conducted in the absence of any commercial or financial relationships that could be construed as a potential conflict of interest.

## Publisher’s note

All claims expressed in this article are solely those of the authors and do not necessarily represent those of their affiliated organizations, or those of the publisher, the editors and the reviewers. Any product that may be evaluated in this article, or claim that may be made by its manufacturer, is not guaranteed or endorsed by the publisher.
